# Two unusual sites of metastases of esophageal adenocarcinoma 

**Published:** 2020

**Authors:** Afshin Amini, Elliot Koury, Zahra Vaezi, Ann Leathersich, Sajid Zafar, Elie Chahla

**Affiliations:** 1 *Department of Medicine, St. Luke’s Hospital, Chesterfield, MO, USA*; 2 *Department of Medicine, Zahedan University of Medical Sciences, Zahedan, Iran*; 3 *Department of Pathology, St. Luke’s Hospital, Chesterfield, MO, USA*; 4 *Division of Gastroenterology and Hepatology, Department of Medicine, St. Luke’s Hospital, Chesterfield, MO, USA *

**Keywords:** Unusual sites, Metastases, Esophageal adenocarcinoma

## Abstract

The most common sites of metastasis for esophageal cancers include the liver, lungs, and bones. We report a rare case of esophageal adenocarcinoma with metastasis to the subcutaneous perianal region as well as to the small bowel. Physicians should consider the possibility of metastasis in a patient with esophageal adenocarcinoma even after the onset of remission. It is essential to examine these patients and maintain a high index of suspicion for possible metastases. Early recognition helps in the accurate staging of the disease and enables the initiation of life-prolonging therapy and achieving meaningful palliation.

## Introduction

 Esophageal cancer accounts for about 4% of all cancers in the United States. It is the eighth most common cancer and the sixth leading cause of cancer-related death worldwide (1). Every year there are an estimated 13,460 new cases and 12,720 deaths attributed to esophageal cancer (2). 

Most cases progress locally, but about 40% of patients can present with metastatic disease, with the most common sites being the liver, lung, and bone (3). Esophageal cancer is an aggressive malignancy with 5-year relative survival rates of 40% in localized disease and 4% when advanced metastasis is present (4). Although the common sites of metastasis are known, studies have shown that esophageal cancer has a unique tendency for expansion to unexpected sites. This has been attributed to several anatomic factors, including the absence of serosal coating and the presence of multiple arterial supplies along with extensive lymphatic dissemination (4). Among these are expansions to the colon or small bowel, as well as cutaneous metastases. Cutaneous involvement is recognized in 1% of cases and is most commonly located in the neck, head, or abdomen, usually involving the skin that overlies the primary tumor. More distant sites may be involved with the most common area being the scalp (5).

Here we report an unusual case of esophageal malignancy with metastasis to the small bowel as well as the perianal tissue. 

## Case Report

An 88-year-old man presented to the hospital with a chief complaint of nausea and vomiting. He had a past medical history significant for recurrent esophageal adenocarcinoma status post-chemotherapy and radiation.

**Figure 1:**
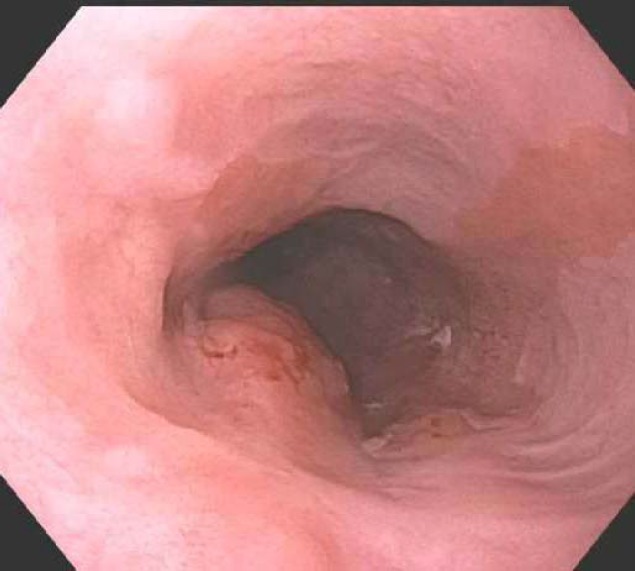
A single 15 mm mucosal nodule with a localized distribution was found in the middle third of the esophagus

Fifteen months before the current presentation, following hospitalization for fatigue and melena, esophagogastroduodenoscopy (EGD) had been performed and noted mucosal changes consistent with long-segment Barrett’s esophagus associated with an esophageal nodule (Figure 1). The nodule was confirmed to be an adenocarcinoma on biopsy and was staged as T3 N1 M0 through endoscopic ultrasonography. Positron emission tomography (PET) scan revealed marked fluorodeoxyglucose (FDG) uptake in the mid-distal esophagus and indeterminate mild activity in prominent mediastinal lymph nodes. 

He received chemotherapy and radiation. Repeat endoscopy four months later demonstrated two linear esophageal ulcers and pathology reported no evidence of malignancy. Eight months later, repeat endoscopy revealed esophageal mucosal changes consistent with long-segment Barrett’s esophagus present in the distal esophagus, which was confirmed by pathology.

**Figure 2:**
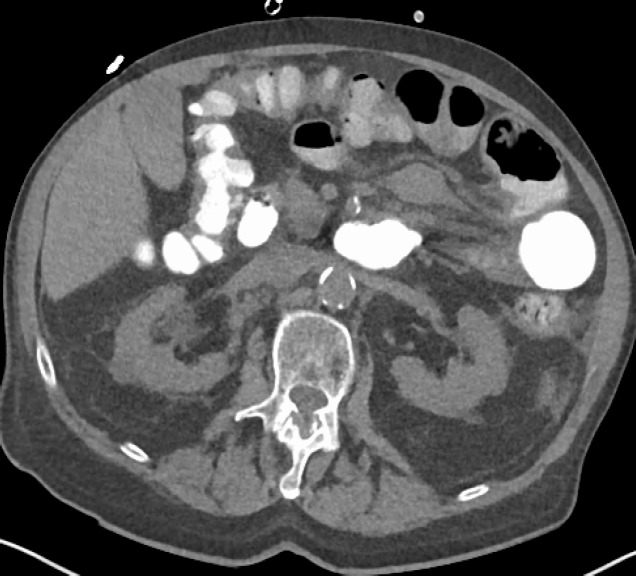
Small mesenteric mass (yellow arrow) which was FDG positive on the previous PET scan

Three months after the last endoscopy, at the current presentation, the patient was admitted for nausea and vomiting. At this time, endoscopy revealed a single 6 mm mucosal nodule in the lower third of the esophagus along with mucosal changes compatible with the established long-segment Barrett’s disease. Histopathology of the nodule was reported as moderately differentiated adenocarcinoma. PET scan indicated focal increased FDG metabolism in the distal esophagus which was worrisome for recurrent malignancy. Furthermore, FDG metabolism was noted at the left supraclavicular, left superior mediastinal, peritracheal, right hilar, and para esophageal nodes suspicious for metastatic disease. A moderately FDG avid nodule in the left upper abdominal quadrant was also noted. This could signal mesenteric adenopathy or non-opacified small bowel. The patient was then started on oral capecitabine treatment. He had noted constipation with his last bowel movement occurring three days prior. A computerized abdominal tomography (CT) showed concerns for small bowel obstruction and small mesenteric mass (Figure 2). 

**Figure 3:**
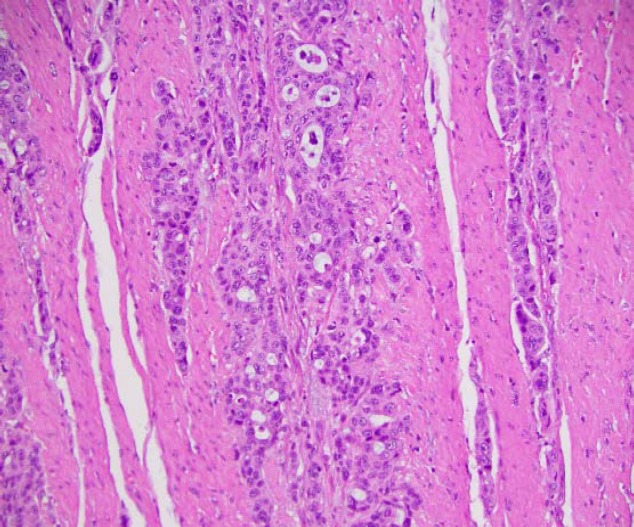
Small intestine, segmental resection (Adenocarcinoma, consistent with metastatic esophageal carcinoma).

**Figure 4:**
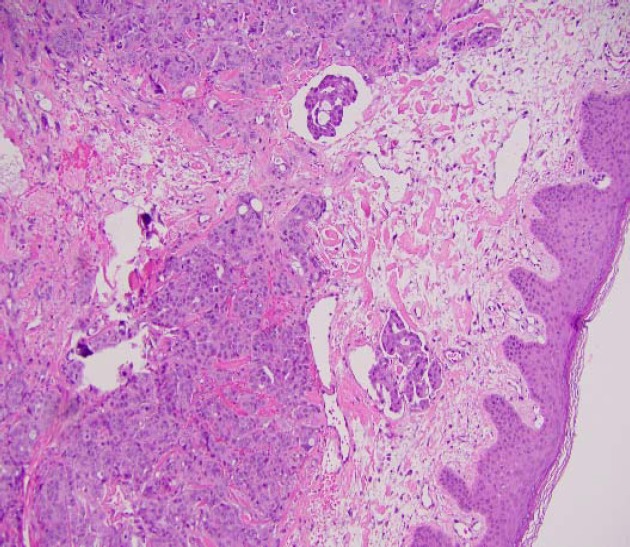
Skin and soft tissue, perianal, biopsy (Metastatic adenocarcinoma, consistent with patient's known esophageal primary).

The primary differential at this point was possible chemotherapy-induced enteritis causing ileus or obstruction. Conservative measures with bowel rest and nasogastric (NG) tube decompression failed to alleviate symptoms. The obstruction persisted, and the patient was initiated on total parenteral nutrition (TPN). Surgical consultation was subsequently obtained, and an exploratory laparotomy was performed, revealing a malignant small bowel obstruction. Small bowel resection was performed with end to end anastomosis, and pathology revealed multiple deposits of moderately differentiated adenocarcinoma consistent with esophageal metastasis (Figure 3). Nausea and vomiting eventually resolved. His constipation and rectal pain worsened, and the rectal exam revealed perianal skin induration. The perianal biopsy was compatible with dermal tissues completely replaced by adenocarcinoma and foci of lymphovascular invasion similar in histology to the known primary cancer. It was, therefore, determined to be a metastatic adenocarcinoma from the known esophageal primary neoplasm (Figure 4). The patient was transferred to hospice care and passed away twelve days after the diagnose of perianal skin metastasis.

## Discussion

Esophageal adenocarcinoma now comprises up to 80% of esophageal cancers in the United States, mainly affecting the non-Hispanic white population (6). The estimated incidence of esophageal adenocarcinoma in patients with Barrett’s esophagus is 0.2% to 2.1% per year (7). 

The most common expected sites of esophageal cancer metastasis include the lymph nodes, liver, lungs, bone, adrenal gland, and the brain (8). Shaheen et al. noted unexpected sites of esophageal cancer metastasis to five main anatomical sites. These include head and neck (42%), abdomen and pelvis (25%), thoracic cavity (17%), extremities (9%), and skin/muscle (7%) (4). 

Cutaneous metastases of esophageal cancer are uncommon and have an incidence of around 1%. These commonly involve the skin of the neck, head, and abdomen. The skin overlying the primary malignancy is commonly involved, but they may also involve distant sites, with the scalp being the most common (9). The most common clinical presentation of these cutaneous metastases is the development of nodules that are usually firm and painless (10). 

The skin manifestation of our case was a painful perianal indurated lesion, which was not reported previously in the literature.

Esophageal adenocarcinoma with metastasis to the small intestine is another rare complication that was present in our case. Until now, only one such case has been reported in the medical literature. Metastatic involvement of the small bowel from any extra-abdominal malignancy is unusual. The involved primary tumors in such cases include malignant melanoma, lung carcinoma, breast carcinoma, skin cancer (non-melanomatous), embryonal myosarcoma, and seminoma (11). Dasari et al. have reported the only case in the literature, which presented as an ileocecal intussusception (12). In our patient, the main manifestation of the small bowel metastasis was intestinal obstruction.

In conclusion, physicians should consider the possibility of metastasis in a patient with esophageal adenocarcinoma even after the onset of remission. It is essential to examine these patients and maintain a high index of suspicion for possible metastases, as recognition can help in the accurate staging of the disease and enables the initiation of life-prolonging therapy and achieving meaningful palliation.

## Conflict of interests

The authors declare that they have no conflict of interest.
